# Older multimorbid patients’ experiences on integration of services: a systematic review

**DOI:** 10.1186/s12913-019-4644-6

**Published:** 2019-11-05

**Authors:** Lilian Keene Boye, Christian Backer Mogensen, Tine Mechlenborg, Frans Boch Waldorff, Pernille Tanggaard Andersen

**Affiliations:** 1 University Hospital of Southern Jutland, Kresten Philipsens vej 15, indgang F, 6200 Aabenraa, Denmark; 2 Focused Research Unit of Emergency Medicine, Department of Regional Health Research, University of Southern Denmark, Odense, Denmark; 3 Emergency Department, University Hospital of Southern Jutland, Jutland, Denmark; 4 Kong Christian X’s Gigthospital, Toldbodgade 3, 6300 Gråsten, Denmark; 5 Focused Research Unit in Rheumatic, Department of Regional Health Research, University of Southern Denmark, Odense, Denmark; 6 Research Unit of General Practice, Department of Public Health, University of Southern Denmark, J.B. Winsløws Vej 9, 5000 Odense, Denmark; 7 Research Unit of Health Promotion, Department of Public Health, University of Southern Denmark, Niels Bohrs Vej 9-10, 6700 Esbjerg, Denmark

**Keywords:** Older patients’, Multimorbidity, Experiences, Integration of services, Continuity of care

## Abstract

**Background:**

Half of the older persons in high-income counties are affected with multimorbidity and the prevalence increases with older age. To cope with both the complexity of multimorbidity and the ageing population health care systems needs to adapt to the aging population and improve the coordination of long-term services. The objectives of this review were to synthezise how older people with multimorbidity experiences integrations of health care services and to identify barriers towards continuity of care when multimorbid.

**Methods:**

A systematic literature search was conducted in February 2018 by in Scopus, Embase, Cinahl, and Medline using the PRISMA guidelines. Inclusion criteria: studies exploring patients’ point of view, ≥65 and multi-morbid. Quality assessment was conducted using COREQ. Thematic synthesis was done.

**Results:**

Two thousand thirty studies were identified, with 75 studies eligible for full text, resulting in 9 included articles, of generally accepted quality.

Integration of health care services was successful when the patients felt listened to on all the aspects of being individuals with multimorbidity and when they obtained help from a care coordinator to prioritize their appointments. However, they felt frustrated when they did not have easy access to their health providers, when they were not listened to, and when they felt they were discharged too early. These frustrations were also identified as barriers to continuity of care.

**Conclusions:**

Health care systems needs to adapt to people with multimorbidity and find solutions on ways to create flexible systems that are able to help older patients with multimorbidity, meet their individual needs and their desire to be involved in decisions regarding their care. A Care coordinator may be a solution.

## Background

The population of those aged 65 and above is increasing, and life expectancy has increased by 30 years worldwide in the twentieth century [1, 2]. With increased age, individuals commonly have two or more chronic conditions, often referred to as multimorbidity [1, 3, 4]. The prevalence of multimorbidity rises with age [5], and nearly 62 and 82% of those aged 65 years and older and aged more than 85 years, respectively, are individuals with multimorbidity [4]. Patients with multimorbidity have a high risk of a reduced quality of life, functional decline, and increased utilization of health care services [4]. Further, their pathway through health care systems can be difficult [6]. They may also need complex care, and they have very specific health care needs [3]. Consequently, health care systems worldwide find it challenging to provide care for these patients [4, 7], which emphasizes the importance of well-coordinated care [8], successful communication, and high interpersonal skills that, when combined, provide a high level of “continuity of care” [9–11]. “Continuity of care” is defined as how patients’ experiences care as coherent and linked over time [11].

Continuity of care is important but may be difficult to establish for people with multimorbidity through the variety of organizations and places involved [9].

Continuity of care as a concept is often presumed rather than defined, and the term is perceived differently [9–12]. Other concepts, for example, “coordination of care”, “continuum of care”, or “integrated care” are commonly used to mean “continuity of care” [11–14]. Put simply, “continuity of care” refers to the way in which patients experience both integration of services and coordination between providers and occurs when elements of care are connected and maintained over time [11]. Nonetheless, “continuity of care” also regards the manner in which patients experience transitions, their relationship with their health care providers and relatives, the transition of information, and consistency of care and personnel [11].

To adapt to both the complexity of multimorbidity and the aging population, health care systems need to improve the coordination of health care services [1, 2, 4]. Understanding how to create health care systems from the perspective of multimorbid patients aged more than 65 years may lead to improvement in health care services and consequently facilitate provision of better quality of life and reduction of functional disabilities among these individuals [4, 15]. Since the existing literature perceives the concept of “continuity of care” differently, we consider it relevant to conduct a systematic review of the literature concerning older people over the age of 65 with multimorbidity and their experiences on integration of health care services. By using experiences on integration of health care services as a simple version of “continuity of care”, we believe we are able to cover “continuity of care” and the aspects made in the different presumptions of “continuity of care”. Therefore the objectives of the present review are to synthesize the manner in which older patients with multimorbidity experience the level of integration of health care services and to identify barriers to continuity of care.

## Methods

We followed the PRISMA guidelines for systematic review in this review [16].

### Literature search

The first author conducted a systematic literature search in February 2018 in the following databases: Scopus, Embase, CINAHL, and MEDLINE. The first author devised the search strategy in collaboration with a research librarian. The search string was adjusted to each database, using the MeSH, Emtree, or exact major subject heading (MM)/exact subject heading (MH) terms for each database. The following search strings were used:

(Comorbidity [mesh, emtree, MM] OR Multimorbidity [emtree] OR (Multimorbid* or multi-morbid* or comorbid* or co-morbid* or multidisease* or multi-disease* or frail* or vulnerab*) OR ((multipl* or cooccur*or co-exist* or co-occur*) adj3 (ill* or disease* or condition* or syndrom* or disorder* or symptom* or medication* or health*))) AND (Aged [mesh, emtree, MM/MH] OR Aged, 80 and over [mesh, MM] OR Senescence [emtree] OR Very elderly [emtree] OR (old* or elder* or aged or geriatic* or senescence or senior* or senium or centenarian* or nonagenarian* or octogenarian* or genrontolog* or “late life”)) AND (Patient Satisfaction [mesh, emtree, MM] OR Patient attitude [emtree, MM] OR Patient preference [emtree] OR ((Patient*) adj3 (Attitude* or opinion* understanding* or perspective* or satisfaction* or preference* or view* or standpoint* or perception* or experience*))) AND (Continuity of Patient Care[Mesh, MM/MH] OR Patient care [emtree] OR Delivery of Health Care, Integrated [mesh, MM] OR Integrated Health Care Systems [emtree] OR Intersectoral Collaboration [mesh, emtree] OR Cooperative Behavior [mesh] OR Cooperation [emtree] OR “contin* of patient care” OR “Patient Care Contin*” OR “Contin* of Care” OR “Care Contin*” OR “Coordinat* care” OR “multidisciplinary care” OR “multi-disciplinary care” OR “Intersectoral Collaboration*” OR “Intersectoral Cooperation*” OR “Integrated Delivery System*” OR “Cooperative Behavior*” OR “Compliant Behavior*”).

### Study selection

After the literature search, the first author screened all studies twice using the titles and abstracts, with a gap of a week between each screening, using the systematic review management program Covidence [17]. Last author was consulted when in doubt. Next, both authors conducted full-text screenings separately. In case they disagreed on certain studies to be included in the full-text screening, first, these two authors discussed such studies and if they did not arrive at an agreement, then the other authors on the research team read and discussed the articles to arrive at a consensus.

### Inclusion criteria

We included studies that fulfilled the following inclusion criteria:

- The studies had to explore the patients’ viewpoints and address aspects such as their experience, and opinions about transitions, their relationship with their health care providers and relatives, the transition of information, and consistency of care and personnel and the health care system they were navigating within. The study participants had to be patients aged 65 years or older.
- The study participants had to be patients with multimorbidity. If this fact was not explicitly stated, only studies with participants aged 85 years or older were included owing to the high proportion of multimorbidity in this age group [4].

We excluded studies that were:

- not written in English, Danish, Norwegian, or Swedish
- not peer-reviewed, published articles.

### Data extraction

#### Analysis

We included only one quantitative study and therefore we chose to code and analyzed it integrated with the other studies, which were qualitative. Therefore, all results are presented narratively. We analyzed all studies using thematic synthesis [18]. The synthesis consisted of three stages. In the first stage, we coded each article line by line according to its meaning and content about the manner in which older patients with multimorbidity experienced transitions, their relationship with their health care providers and relatives, the transition of information, and consistency of care and personnel, creating several initial codes. In the second stage, we formed descriptive themes to capture the meaning of the initial codes. In the first and second stages, we stayed close to the original findings in the included studies. Creating the descriptive themes offered us the possibility to go beyond the content in the original studies. In stage three, we created analytical themes, which means we used the descriptive themes to find answers to our research questions [18]. We used QSR International’s NVivo11 qualitative data analysis software to conduct the synthesis [19].

#### Quality appraisal

We used the CoreQ checklist for assessment of study quality [20]. In rating the quality of the included study, we focused only on domain 2, Study design (items 9, 10, 11, 12, 14, 15, 16, and 17), and domain 3, Analysis and findings (items 26, 29, and 30). Each study was assigned one point if the items were identified; therefore, a study could obtain 0–11 points, with 11 being the highest score, thus indicating highest quality. We rated the quality high if the score was 8 to 11, medium if 4 to7 and low if 0 to 3. We only included articles scoring 4 or higher. The first author conducted the quality assessment, and last author reviewed the results.

## Results

### Literature search

**Fig. 1:**
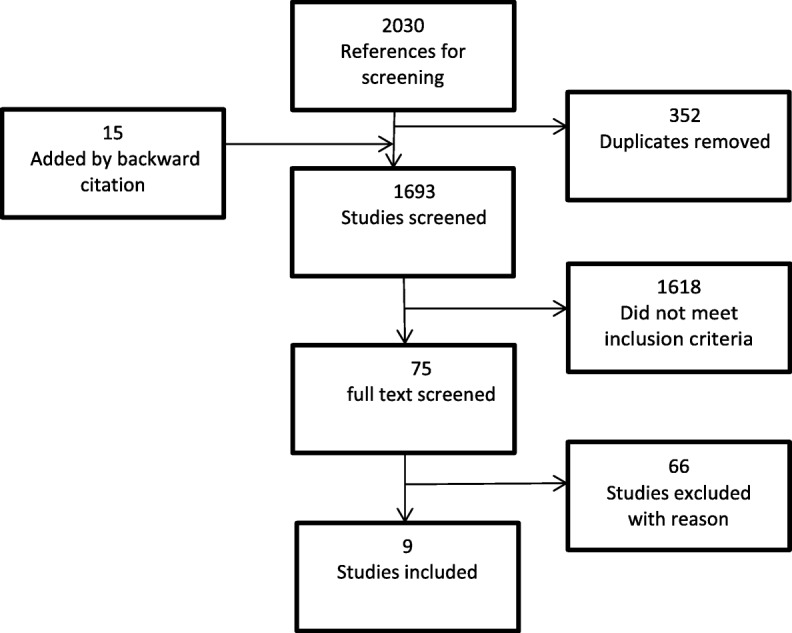
PRISMA flow diagram

We identified a total of 2030 studies, 310 from Embase, 1282 from MEDLINE, 258 from Scopus, and 180 from CINAHL. An additional 15 records were added after identification by backward citation. After removal of 352 duplicates, 1693 studies remained for title and abstract screening. After title and abstract screening, we excluded 1618 studies and found 75 studies eligible for full-text screening. We excluded most studies because they did not meet the age criterion and the patient’s viewpoint criterion. After the full-text screening, nine articles remained for quality assessment and final synthesis, as shown in the PRISMA Chart in Fig. 1. All these studies are on Western countries and are published between 2008 and 2017.They represent a broad variety of patient groups. The included papers focus on the patients experience with a transition between primary and secondary care setting or vice versa or on how the patients experience the health care system in general. Most interviews were conducted after the patient was discharged or had visited their general practitioner. Characteristics of the selected studies [21–29] are presented in Table 1.

**Table 1:** Overview of the included studies

1_st_ Author, year	Country	Study design	Time for data collection	Setting	Multimorbidity	Type of participants	No. of participant (%men)	Age *mean +/− sd*, median, {range}, (age-groups)	Objectives	Quality rating: repor-ted/items
Andreasen, 2015 [22]	Denmark	Interviews	1 week after discharge	Primary to secondary	Tilburg Frailty Indicator + comorbidity	Acutely admitted frail elderly	14 (50)	*80.6,* {69–93}	“was to explore how the frail elderly experience daily life 1 week after discharge from an acute admission to the hospital”	11/11
Arendts, 2015 [21]	Australia	Interviews	When resident were able to do the interview	Secondary to primary	Unclear*	Residents of Residential Aged Care facilities (RACF)	11 (18)	*88*	“to capture and interpret the perspectives of three important decision-making groups concerning the transfer of residents from RACF to Emergency department; to understand how the perspectives of these converge and diverge; and to explore shared decision-making and the extent to which there was delegation of transfer decisions to others”	8/11
Bayliss, 2008 [23]	USA	Interviews	Unclear	Secondary	Three target conditions (diabetes, depression, osteoarthritis) + self-reported condition	Members of a not- for-profit Health Maintenance Organization	26 (50)	(65–84)	“was to explore patient perspectives on components of ‘best’ Processes of care for persons with multiple morbidities in order to inform the development of future interventions to improve care”	8/11
Butterworth, 2014 [24]	United Kingdom	Interviews	Unclear	Secondary	14 participants had one or more chronic diseases	Registered with surgery for at least 6 month	20 (45)	(65–74), (75–84), (85–94)	“to investigate the association between older patients’ trust in their general practitioner and their perceptions of shared decision-making.”	8/11
Foss, 2011 [25]	Norway	Face to face questionnaire	2–3 weeks after discharge	Primary to secondary	Unclear*	Patients discharged from hospitals	254 (31.5)	*86.9 +/− 4.9*	“was to describe older hospital patients’ discharge experiences concerning participation in discharge planning”	10/11
Gabrielsson-Järhult, 2016 [26]	Sweden	Observations and discharge meeting material	Before discharge	Primary to secondary	Unclear*	Admitted to hospital and about to be discharged	27 (37)	**81**	“was to explore older people’s concerns about their needs as expressed in a discharge planning meeting at a hospital”	10/11
Gill, 2014 [27]	Canada	Interviews	Unclear	Primary to secondary	Two or more chronic conditions	Patients from a family health team	27 (56)	*82.3 +/− 7.7*	“was to explore the challenges experienced by 27 patients-caregivers-family physician triads in an attempt to capture a full understanding of their health system experience and to illuminate where system improvements are most needed for managing multimorbidity”	11/11
Neiterman, 2015 [28]	Canada	Interviews	2–5 weeks post discharge	Primary to secondary	Lace score 10 or higher	Patients discharged from acute care hospital	17 (58)	(70–89)	“was to understand how patients and their caregivers experienced the transition to community and which barriers and facilitators they identified on their way to recovery”	11/11
Sheaff, 2017 [29]	United Kingdom	Interviews	May 2012–November 2013	Primary to secondary	Two or more specified chronic conditions	Patient who had been admitted within a year and who had received care from 2 separate healthcare services	66(NA)	*78*, (65 or older)	“was to analyze what information was changed or lost in communication between clinicians and a group of frail older patients in England, and some implications for care coordination and continuity”	6/11

*Included because we know that among those aged more than 85 years, 82% are patients with multimorbidity

### Quality assessment

We included all nine selected articles in the final synthesis, and rated these from medium to high quality. The quality rating is shown in Table 1.

### Thematic synthesis

**Fig. 2:**
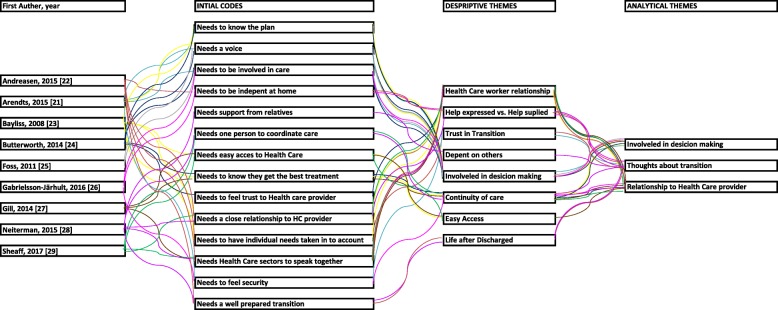
Detailed coding tree of the thematic synthesis

The line-by-line coding resulted in 16 initial codes. In creating the descriptive themes, we searched all initial codes for similarities and differences and 10 descriptive themes emerged from this process. All 10 themes were centered on the experiences of the older multimorbid patients, and all contributed to three analytical themes. The three main issues that emerged from the studies on ways in which the older multimorbid patients experienced integrations of health care services were: (1) “Involved in the decision-making,” (2) “Thoughts about transition,” and (3) “Relationship to health care providers.” The main themes overlapped each other, although only one [26] of the included studies contributed to all themes. The overlapping issues in the main themes are *communication* between the health care providers and the older multimorbid patient and *granting individual needs* of these patients. The coding tree is shown in Fig. 2.

### Continuity of care

#### Involved in decision making

We found through the synthesis that the older multimorbid patients felt that the level of integration of services and coordination between health care providers was a success when they experienced being involved in the decision process and they felt listened to, and acknowledged for their awareness, about their own needs [23, 24, 26]. The patients who had been involved in the decisions process felt more trust in their health care provider [24]. When the multimorbid patients expressed that they had not being involved in the decision process regarding their own care [25], integrations of services were unsuccessful and it made them feel less trust towards their health care providers [21].

##### Thoughts about transition

This theme captured that the older multimorbid patients experienced the level of integrations of services as successful when they had relatives to rely on in a transition [28] and when they felt a sense of security on transfer from an aged-care facility to an emergency department, because they felt safer at the latter [21]. When the integration of services and the coordination between health care providers did not succeed, these patients felt that they were discharged too early, and that made them feel insecure [22, 28]. This finding also applied in situations in which they were discharged with no help from anyone other than their relatives [22]. When at home, these patients also experienced the feeling of being treated as an object by their health care providers [22] and that they did not receive the help they needed or were receiving help but not to the extent they perceived to be adequate [22, 26]. In particular, patients without relatives expressed their inability to assume responsibility for their own care [28]. They wanted to be listened to about their wishes for help in their own home [22]; experiences of disorganized post-discharge care were not uncommon [28].

##### Relationship to health care providers

These older multimorbid patients experienced integrations of services as successful when they had convenient access to their health care providers [23] but felt frustration at times, especially when they had to wait for long periods [23, 27, 29] and when physicians only examined one health issue per visit [29]. Concurrently, the patients did not want to bother their physicians unduly [20, 22]. The older multimorbid patients also needed to know that their health care provider as well as their general practitioner acted in patients’ best interest [24].

Integration of services was also successful when the patients had a care coordinator to help them prioritize the demands that their multiple conditions imposed upon them and when the coordinator helped them keep track appointments and their health conditions [23, 28, 29]. The care coordinator could be the GP, and it was important for the patient to visit the same GP to obtain continuity [24, 29]. These patients also perceived continuity of care when they felt listened to and the care was finalized from their point of view [24]. When the integration of services and the coordination between health care providers did not succeed, it was a barrier to obtaining continuity of care.

## Discussion

### Principal findings

This review shows that multimorbid patients (aged 65 years or older) in various settings experience both successful and unsuccessful integration of health care services. It was successful when the patients felt listened to on all the aspects of being individuals with multimorbidity and when they obtained help from a care coordinator to prioritize their appointments. However, they felt frustrated when they did not have easy access to their health providers, when they were not listened to, and when they felt they were discharged too early. These findings underpin the need for health care systems to adapt to the growing older population and people with multimorbidity and to find solutions on ways to create flexible systems that are able to help older patients with multimorbidity, meet their individual needs and their desire to be involved in decisions regarding their care.

### Continuity of care

The thematic synthesis found three main themes, “*Involved in decision making,” “Thoughts about transition,”* and *“Relationship to health care providers”*; the overlapping issues were *communication* and *granting individual needs* for the older multimorbid patients.

From the themes “Involved in decision-making” and “Thoughts about transition,” we know that these patients did not feel listened to and that they were not always participating in their discharge plan. This finding is not new knowledge [12], but it indicates that the health care systems still need improving and must focus on creating a better communication flow for older multimorbid patients. Studies have also found that involving patients in their discharge solutions and providing tailored individual plans may result in a small reduction in the length of hospital stay and reduce the probability of readmission rate within the 3 month [30]. Further, these patients have more trust in their health care provider when they are involved in the decision process. This finding is very interesting because it indicates that the more the patients’ involvement, the more their trust in the health care system. We know that patients experience continuity when they feel trust in their health care provider and distrust when they experience gaps in their care [31–34], and it raises the following question: Does trust via patient involvement create a high level of integration of services and thereby a high level of “continuity of care”? The findings in this review indicate that it does.

Multimorbid patients have a high use of health care services [6], and as we know from the theme “Relationship to health care providers,” it can be difficult for them to have easy access to their health care providers. Further, from “Thoughts about transition,” we know that older multimorbid patients also request that their individual needs be considered when receiving home care from the health care system and that they do not always fit into the “standard” care. Certain studies have stated that prioritizing the demands that matter the most to these patients, by listening to them, is key to providing them the most appropriate care [35]. This finding indicates a need for flexible solutions. The need to create flexible solutions is one finding in the theme “Relationship to health care provider,” in which we described these patients’ desire to have a care coordinator to take care of their complex situation. Health care systems may be able to create a high level of continuity of care, if the older multimorbid patient has one person who is able to coordinate their care [36]. Studies have also stated that having a care coordinator is most important when a patient is multimorbid [31]. Being older individuals with multimorbidity places these individuals in a position where they may have limited resources [37]. Being an individual with multimorbidity is a complex situation, as findings from the three themes show, and as found in several other studies [3–6]. This fact raises the following question: How should the health care systems be changed to create a high level of integration of services and avoid the experiences that are a barrier to continuity of care?

### Strengths and weaknesses of the study

The strength of this review lies not only in the systematic approach we used to find the included articles but also in the synthesis of the experiences of the integration of health care services from the older (≥ 65) multimorbid patients’ perspectives. We used thematic synthesis to address the manner in which these patients experience the level of integration of health care services and to identify barriers to continuity of care. Certain researchers may argue that it is not possible to synthesize qualitative research when the individual studies are decontextualized and the themes identified in one setting might not be applicable to other settings [18, 38].However we, as well as other researchers [18], believe that by checking that we did not interpret themes into settings to which they did not belong, we grounded the text in the context in which it was constructed. Other strengths are that this review contributes to knowledge in this area from the patients’ perspectives, and all the studies included in this review are of medium to high quality based on design, analysis, and findings.

A weakness in this review is that it is a challenge performing a search on both individuals’ “experiences” and “multimorbidity.” The term multimorbidity is used in various ways, as is the term continuity of care. Therefore, we structured the search string to catch this variety. We believe we covered as much of the literature as possible by including the most-used terms for both multimorbidity and continuity of care. We acknowledge that we found 15 studies by backward citation, but none of them was included in the final review. The included studies differ in characteristics, which can be viewed as a limitation. However, we do believe that by including studies conducted across different settings, we can achieve a higher level of abstraction [38]. Another limitation is that only one author conducted the screening of the title and abstracts, which we mitigated by blind screening of the title and abstracts twice, 1 week apart.

## Conclusions

This review adds that it is necessary to conduct high-quality research on methods of considering the individual needs of multimorbid patients above the age of 65 years in developing health care systems. This review found consistent evidence that these older patients wish to have their individual needs taken into consideration in their care plan and they want to be listened to on all aspects of their care. Thereby, they also felt higher trust towards their health care providers. It may also be essential for these patients to have a care coordinator, and health care systems as well as the patients will benefit from having such coordinators. Appointing coordinators may even prevent these patients’ unsuccessful experiences of not being involved in the decision process, their feelings of being discharged too early and of not receiving sufficient help after discharge, and their lack of easy access to health care providers. Thus, it would provide multimorbid patients the feeling of being listened to and having their individual needs considered and remove the barriers to achieving continuity of care. Potential research directions are to determine ways to integrate the older multimorbid patients’ individual needs into a trustworthy health care system and to create flexible solutions in a standard system that includes differences and more vulnerable older patients without a strong social network and support.

## Data Availability

The dataset used and analyzed during the current study, including a list of the excluded articles, are available from the corresponding author on reasonable request.

## Abbreviations

MH: Exact subject heading, search term in Cinahl database
MM: Exact major subject heading, search term in Cinahl database
RACF: Residential Aged Care facilities
